# One Health and the Exposome: providing insights for wildlife health and reproduction

**DOI:** 10.1016/j.xfre.2025.02.001

**Published:** 2025-04-15

**Authors:** Mary Ann Ottinger, Cullen Geiselman

**Affiliations:** a Department of Biology and Biochemistry, University of Houston, Houston, Texas; b The Cullen Trust for Health Care, Houston, Texas

**Keywords:** One Health, Exposome, reproduction, wildlife, lifetime reproduction

## Abstract

Although anthropogenic activities are often responsible for the loss of biodiversity, humans are not immune from the adverse consequences of diminishing wildlife populations and the resulting declines in ecosystem resilience and health. The adverse effects of the changing environment and specifically their influence on the reproduction and overall fitness of wildlife and humans alike are pertinent to the topic of this special issue. This article will consider environmental factors that influence wildlife health and biodiversity and affect human health and discuss approaches to evaluate status, monitor change, and develop interventions. Examples and further reading will be provided as well as a case study illustrating the utility of the One Health and Exposome conceptual frameworks as approaches for interventions and restoration. Finally, exposomics provides an emerging diagnostic for wildlife health and exposure to environmental stressors that should be developed and optimized for use in the field to assess the condition and risk for wildlife populations.

Wildlife throughout the globe experiences a suite of environmental stressors, many of which are caused by human endeavors ranging from road building to industrial agriculture. As a result, there have been dramatic declines in wildlife populations varying with region, environmental stressors, and the animal’s evolutionary history (1, 2). The Living Planet Index, composed of data compiled annually since 1970 from 20,000 distinct populations of mammals, birds, reptiles, amphibians, and fishes, estimates these aggregate declines to be approximately 68% (Fig. 1) (3). For the animals that remain, stress from diminishing resources, toxins, and other anthropogenic elements can adversely affect their health and fitness, resulting in low reproductive output further exacerbating the declines (4, 5, 6, 7, 8, 9, 10, 11, 12, 13). Tackling the myriad threats and stressors wildlife face in a piecemeal fashion has not halted or reversed many of these declines, especially for long-lived, slow-reproducing species. We propose using the new combined One Health-Exposome conceptual framework to address threats to wildlife in a more holistic manner (1). Here, we address the plight of declining African vulture populations using this combined framework as a case study.

**Figure 1:**
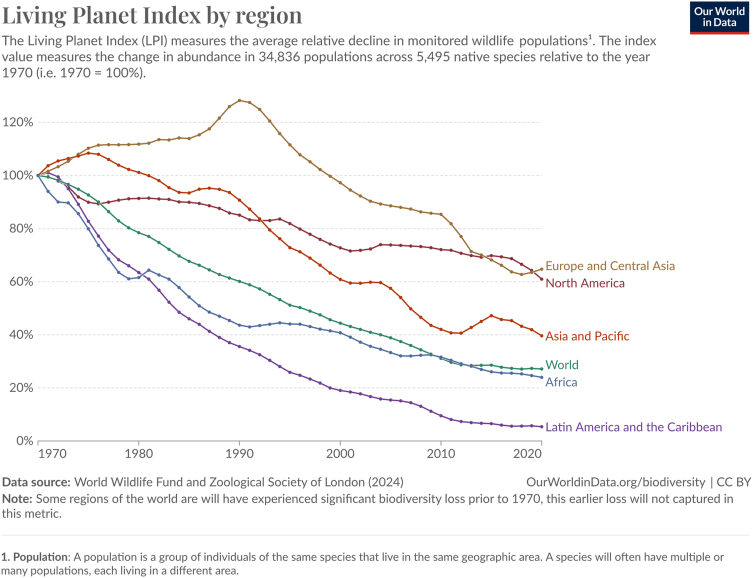
Data showed the percentage decline in wildlife populations over time in large global areas, with the world decline also shown (from Our World in Data, downloaded on November 15, 2024).

## One health and the exposome conceptual frameworks

**Figure 2:**
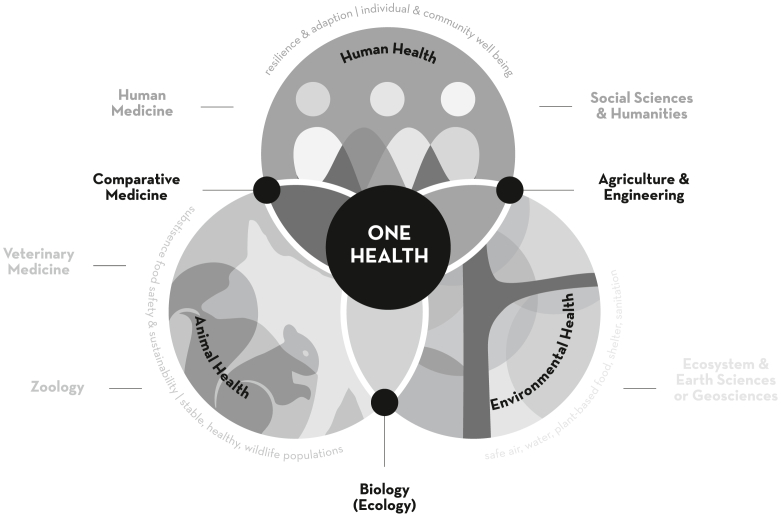
The One Health concept embraced the interrelation of humans, animals, and environment. Given this breadth, many disciplines were brought together under this framework. (From Author Ottinger Geiselman (1). Reprinted by permission of the publisher.)

**Figure 3:**
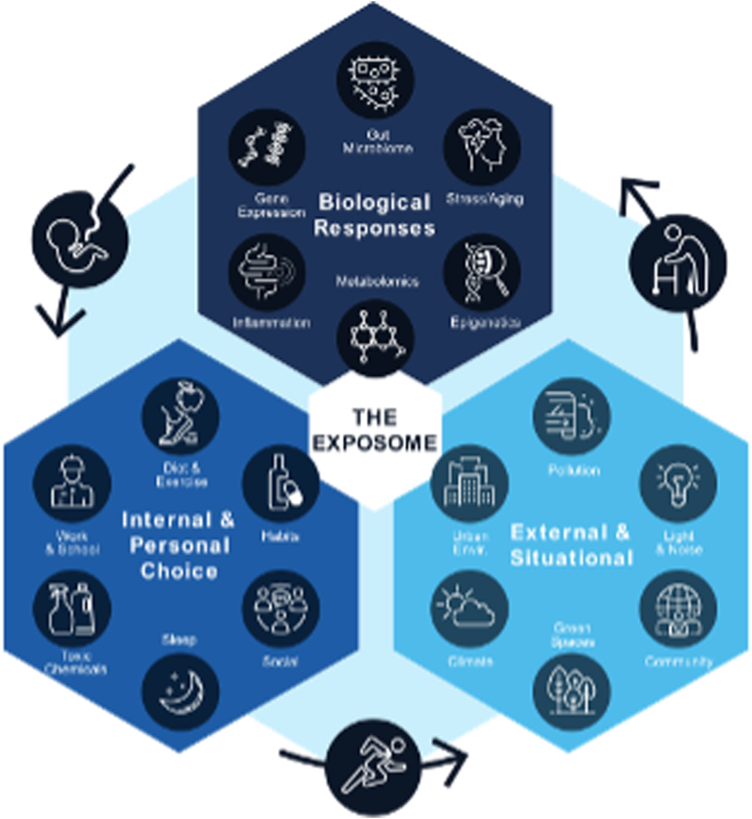
The Exposome concept espoused that an individual’s exposome incorporated impacts from internal conditions and personal choice, external and situational exposures, and the biologic responses to these over the course of a lifetime. (From Author Ottinger Geiselman (1). Reprinted by permission of the publisher.)

One Health and the Exposome are 2 conceptual frameworks that may aid in developing solutions to local or regional declines in wildlife although they differ greatly in focus and scope. The One Health concept stresses the interrelatedness of all living things and how the health of humans is linked to that of wildlife and the environment (Fig. 2). It provides a structured approach for building multidisciplinary teams to address crises at their root causes and historically addressed disease spillover events from wildlife to humans (Fig. 3) (1, 14). Over time, the concept has broadened to include many types of environmental issues spanning human-wildlife conflict to environmental disasters (15, 16).

**Figure 4:**
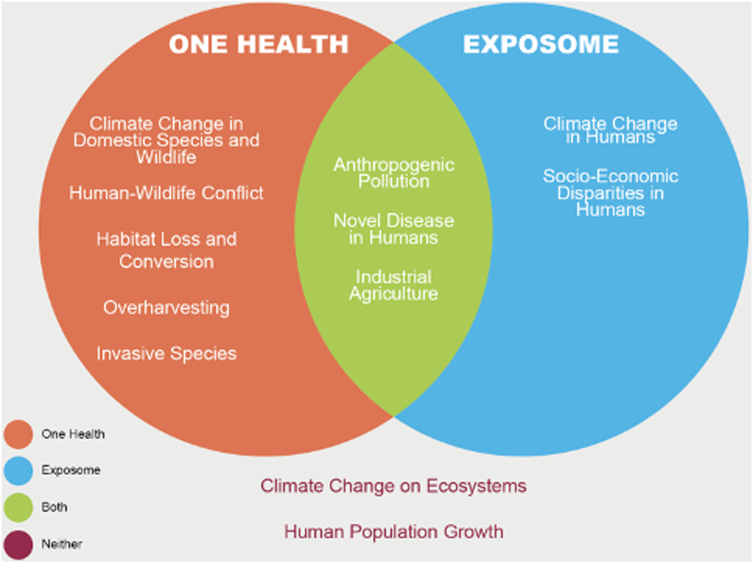
The One Health and the Exposome concepts overlapped in confronting challenges to human, domestic species, wildlife, and ecosystem health. (Adapted from Author Ottinger Geiselman (1). Reprinted by permission of the publisher.)

In contrast, the Exposome concept grew out of biomedical research as a counterpoint to the Genome, acknowledging the link between environmental exposures and gene expression (17). It addresses the effects of exogenous exposures on the health of an individual at a point in time or over their lifespan (Fig. 3). It has recently broadened to include exposures related to socioeconomic factors (18). Although it has not been applied to wildlife, it has great potential because wild animals suffer from toxins and environmental stressors in similar ways to humans. Ottinger and Geiselman (1) provide an extensive review of both concepts and outline where the 2 currently overlap in their utility to address certain issues (Fig. 4).

**Figure 5:**
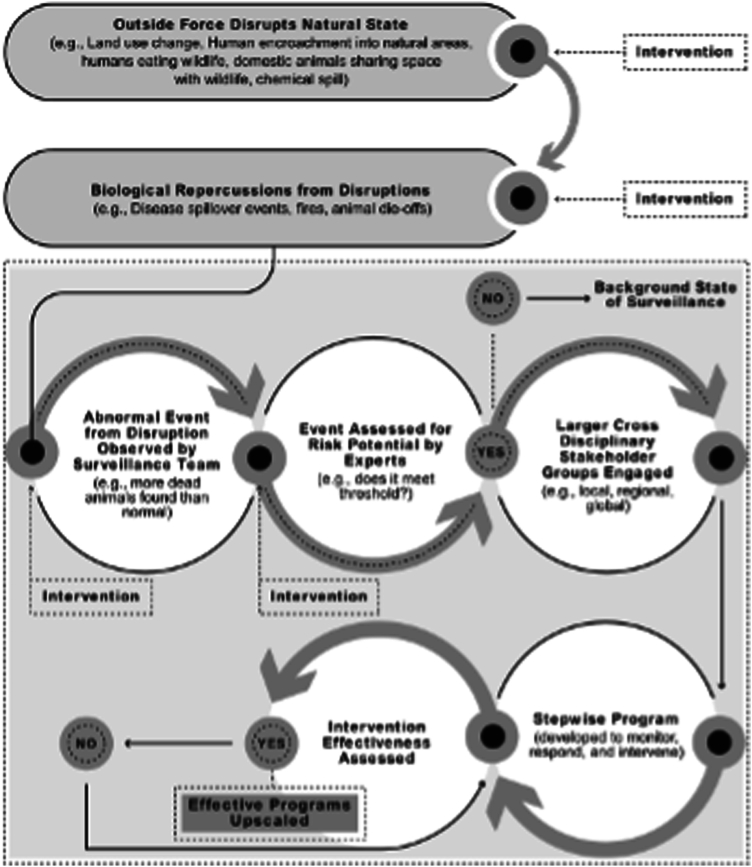
The One Health conceptual framework was used for identifying, assessing, and implementing an actionable program. The process was as follows: (1) external disruption to the current natural state resulted in (2) biologic repercussions, such as disease spillover, (3) that were witnessed as abnormal events by the surveillance team; (4) experts assessed whether the event reached a risk threshold; (5a) if yes, a cross-disciplinary team develops a stepwise program to monitor, respond, and/or intervene; (5b) if no, background surveillance continued; (6) effectiveness of intervention from 5a was assessed; (7) if effective (yes), the program was upscaled; and (8) if not effective (no), the program was revised. (From Author Ottinger Geiselman (1). Reprinted by permission of the publisher.)

Using the One Health framework (Fig. 5) provides the opportunity to identify and intervene in a process that exerts damage to wildlife and affected ecosystem(s). It is notable that there should be a “trigger” that often is in the form of an environmental disaster, such as chemical spill poisoning wildlife causing a die-off.

**Figure 6:**
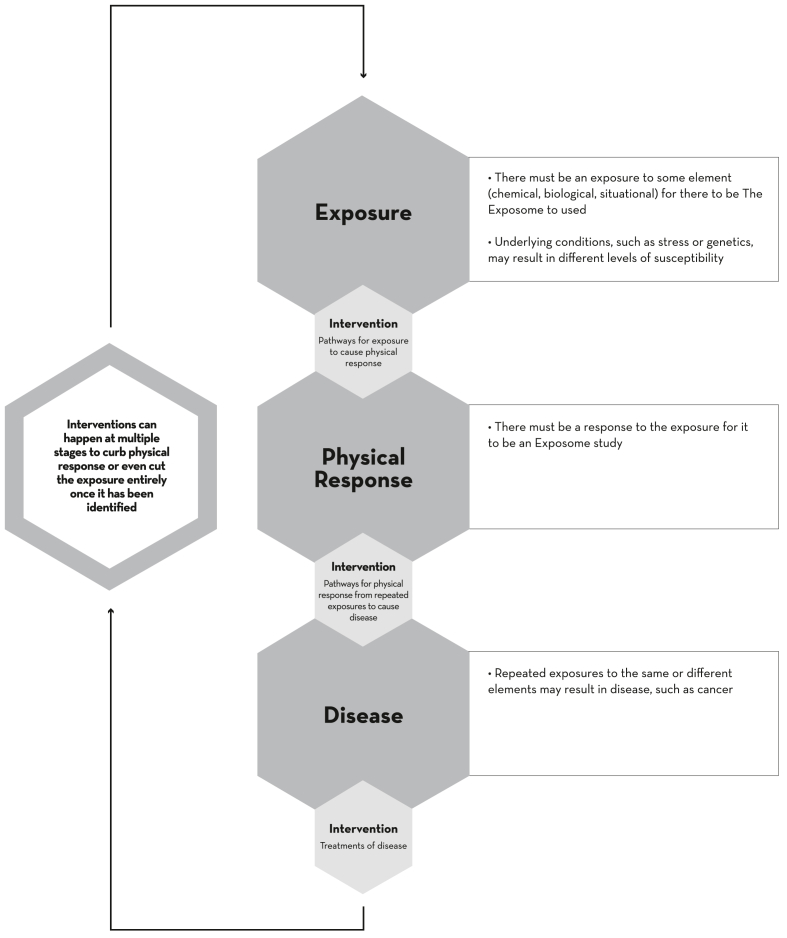
The Exposome conceptual framework provided a stepwise functional path for assessing and implementing actions, with potential for intervention at multiple steps in the process. (From Author Ottinger Geiselman (1). Reprinted by permission of the publisher.)

The Exposome approach engages a framework that is triggered by 1 or more interacting stressors at various stages of the life cycle (Fig. 6). Accordingly, the emerging field of exposomics, a suite of physiological measures associated with exposure to environmental chemicals and other stressors, provides valuable indicators and diagnostics of exposure. The key questions are whether the combined One Health-Exposome framework can better address threats and stressors to wildlife than current approaches and whether exposomics can provide reliable metrics of environmental exposures and stressors to monitor overall health of a population.

## A case study example: african vultures

Eleven species of Old World vultures inhabit Africa, and their populations have declined by 80%–97% over the last 50 years under pressure from land use change, deliberate and unintended poisoning, environmental chemicals, and human encroachment and harvesting for cultural practices (19, 20, 21). These long-lived birds, which consume carrion putting them at the top of the food chain, may fledge only 1–2 chicks/breeding cycle further exacerbating the rate of population decline. Ongoing monitoring programs provide critical data on lifetime productivity and survival, monitor movement in relation to resources, and, with associated metadata, provide valuable information for conservation programs. The International Union for Conservation of Nature produced the Multi-species Action Plan to Conserve African-Eurasian Vultures that promotes range-wide assessment, monitoring, and other actions (22). The key to making forward progress is to coordinate these programs. In the following, we illustrate using the combined One Health-Exposome framework to address the multifaceted issues facing African vultures.

**Figure 7:**
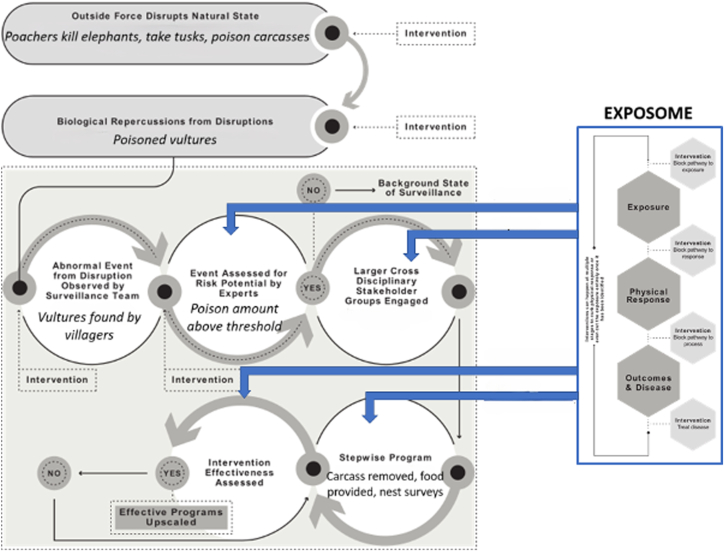
The combined One Health-Exposome framework addressed the vulture die-off. (modified from Author Ottinger Geiselman (1). Reprinted by permission of the publisher.)

In this scenario, poachers have shot and killed elephants, cut out their tusks, and poisoned the carcasses so their locations will not be given away to game wardens by circling vultures. Here, the One Health approach can help to untangle the complex interrelationships among the factors at play in an integrated manner, thereby laying the groundwork for developing an operational One Health framework. The Exposome, which can easily nest inside of this framework, is more attuned to the molecular and biologic pathways to diagnose exposures as indicators and mechanisms of effect (Fig. 7). As shown in Figure 7, the outside force disrupting the natural state is the poaching of elephants and poisoning of their carcasses. The biologic repercussions from this are poisoned vultures. This abnormal event (dying or dead vultures) is found by villagers and reported to game wardens, who take samples to be analyzed by wildlife veterinarians. The samples are positive for a pesticide, such as dieldrin or malathion, in amounts determined by exposomics to be above the recommended threshold, and a multidisciplinary team of experts and local stakeholders is assembled. They develop a stepwise program to remove the poisoned carcasses, test vulture populations for contamination, surveil nesting sites for fragile eggs (outcome of poisoning), provide alternate food sources, and monitor for additional changes while increasing surveillance for poachers. Of note, exposomics is used in multiple stages of this process.

This simple example reveals the utility of nesting exposomics within the One Health framework to combat anthropogenic threats to wildlife. In particular, the Exposome framework should be expanded beyond humans to include other animals to build out exposomics with its valuable indicators and diagnostics of exposure for wildlife. Despite the utility of this combined framework to address local and regional challenges, it begins to breakdown when addressing wildlife trade driven by global markets where the source of the deleterious behavior is far removed from where its effects are felt.

## Declaration of Interests

M.A.O. reports royalties from Elsevier press. C.G. has nothing to disclose.
